# EPHA2 promotes triple-negative breast cancer progression by suppressing pyroptosis via the AKT/PI3K/mTOR pathway

**DOI:** 10.3389/fonc.2025.1620122

**Published:** 2025-08-22

**Authors:** Xiaoying Huang, Li Na, Qingkai Han, Qilun Liu, Ligang Wu

**Affiliations:** ^1^ General Hospital of Ningxia Medical University, Yinchuan, China; ^2^ Biobank, General Hospital of Ningxia Medical University, Yinchuan, China; ^3^ Ningxia Medical University, Yinchuan, China

**Keywords:** EPHA2, pyroptosis, SLC7A11, AKT/PI3K, triple-negative breast cancer

## Abstract

**Background:**

Breast cancer (BRCA) is the most prevalent cancer in women, with triple-negative breast cancer (TNBC) accounting for 15-20% of cases. TNBC is associated with higher rates of metastasis, recurrence, and poorer prognosis, underscoring the urgent need for new diagnostic and therapeutic strategies.

**Methods:**

In this study, multiple public online platform, including UCSC Genome, UALCAN, Kaplan Meier plotter, DepMap and Single Cell Portal were used to detect the expression of EPHA2 in TNBC. Cell Counting Kit-8 (CCK-8) and transwell assays were conducted to assess proliferation and invasion. KOBAS bioinformatics, transmission electron microscopy (TEM), ELISA, western blot and quantitative real-time PCR experiments were employed to detect the association and effects of EPHA2 on pyroptosis in BRCA.

**Results:**

EPHA2 was highly expressed in TNBC, and showed a negative correlation with survival. Single-cell analysis indicated that EPHA2 was mainly expressed in stromal and epithelial cells, particularly within TNBC compartments. Furthermore, we found that EPHA2 knockdown inhibited cell proliferation and invasion, and induced pyroptosis, as evidenced by increased level of pyroptosis-related protein (IL-18, IL-1β) and characteristic morphological changes. Moreover, a relationship between EPHA2, pyroptosis, and the AKT/PI3K pathway was established and confirmed. Additionally, we observed a decreased expression of ferroptosis-associated marker named SLC7A11, suggesting that this transporter may mediate the effects of AKT inhibition on pyroptosis.

**Conclusions:**

In summary, our findings illuminated the dual roles of EPHA2 in TNBC, influencing both tumor progression and cell death pathways. We hypothesize that SLC7A11 serves as a key regulator of pyroptosis in the context of EPHA2 and AKT/PI3K signaling. These insights underscore the potential of targeting these pathways in developing therapeutic strategies for BRCA treatment. Further investigations into the mechanisms underlying SLC7A11’s roles could enhance our understanding of its therapeutic implications.

## Introduction

Breast cancer (BRCA) is the predominant malignancy in women, with the number of cases continuing to rise each year (1). Triple-negative breast cancer (TNBC) is characterized by the absence of estrogen receptors (ER), progesterone receptors (PR), and human epidermal growth factor receptor-2 (HER2) (2). TNBC accounts for 15-20% of BRCA diagnoses and lacks clinically validated therapeutic targets. This subtype is associated with a higher rate of metastasis and recurrence, as well as a poorer prognosis compared to other BRCA subtypes (3, 4). Chemotherapy remains the primary treatment for TNBC, and the negative immunophenotype indicates that patients do not benefit from targeted therapies such as trastuzumab and tamoxifen (5, 6). Therefore, there is an urgent need for the discovery of new diagnostic and therapeutic targets in TNBC.

Ephrin receptors (Eph) belongs to the family of receptor tyrosine kinases (RTKs), comprising 14 cell-bound members. The RTKs are divided into two groups, ephrin-A (EPHA1–EPHA8 and EPHA10) and ephrin-B classes (EPHB1–EPHB4 and EPHB6) (7). There is an extracellular receptor-binding domain (RBD), binding RBD of the ephrin ligands, in the N-terminus of ephrin-A and ephrin-B. The Eph receptors have catalytic and non-catalytic functions (8). The EPH/ephrin system is critical for human physiology, and its dysregulation has been implicated in a variety of human diseases (9). As reported, the aberrant expression of Eph receptors has been found in many cancers and contributes to various tumorigenic processes (10) However, recent studies have highlighted that Eph receptors play a dual role, acting as both tumor suppressors and promoters of tumor progression (11, 12). The EPHA2, EPHA3 and EPHA4 have been found correlated with a variety of cancers, including lung carcinoma, prostate carcinoma, colon carcinoma, pancreatic carcinoma, ovarian carcinoma, thyroid carcinoma, tongue carcinoma, hepatocellular carcinoma, glioma and melanoma (13–18). Especially, EPHA2 regulated tumor microenvironment (TME) through both ligand-dependent and ligand-independent mechanisms. Cell-to-cell contact serves as the primary mechanism for regulating both canonical and non-canonical signaling pathways (19, 20). The function of EPHA2 is complex in different cancers.

Over the past three decades since 1990, strategies targeting EPHA2 -ephrin A1 signaling axis has been developed and several clinical studies are currently in progress. The mechanisms of action of these strategies include inhibiting EPHA2 activation, reducing EPHA2 expression, enhancing EPHA2 degradation, employing EPHA2-targeted immunotherapy, and facilitating EPHA2-mediated drug delivery (9, 14, 21–25). However, they have demonstrated limited success, highlighting the urgent need for further research to elucidate the comprehensive biological roles of EPHA2. The roles of EPHA2 in cancer cell proliferation, migration and stemness have been well studied (26–30). This study demonstrates that EPHA2 is involved in the pyroptosis of breast cells by modulating the AKT/PI3K/mTOR signaling pathway, and regulating the expression of SLC7A11 associated with ferroptosis through this signaling cascade. These data indicate that EPHA2 plays an important role in cancer cell death (31).

## Materials and methods

### Bioinformation analysis

The EPHA2 expression, percent survival was analyzed using UALCAN web server, UCSC Genome and Kaplan Meier plotter (32, 33). The EPHA2 protein expression in different breast cancer cell lines was analyzed using DepMap portal (34). The expression of EPHA2 at the single-cell level was analyzed using the GSE176078 dataset in conjunction with the Single Cell Portal (35, 36). KOBAS (http://bioinfo.org/kobas) was used to assess pathway enrichment of differentially expressed genes (DEGs) (37).

### Cells and regents

MDA-MB-231, MDA-MB-468, BT-20, HEK293T and MCF-10A cells were purchased from ATCC and preserved in our lab. SUM159 cell was purchased from EK-bioscience. The cells were cultivated in medium specified in the instructions. The DMEM, RPMI 1640, Fetal bovine serum (FBS), penicillin-streptomycin solution and PBS are purchased from Fisher Scientific Co., Ltd (Shanghai, China). Antibodies targeting NLRP3 (D4D8T), Cleaved Caspase-1 (Asp296), Cleaved Gasdermin D (E7H9G), PI3 Kinase p85, phospho-PI3 Kinase p110 beta (Ser1070), Akt1 (C73H10), and Phospho-Akt1 (Ser473) (D7F10) were purchased from Cell Signaling Technology (CST, Danvers, MA, USA). Antibodies targeting SLC7A11 and EPHA2 were purchased from Boster Biological Technology.

### Lentivirus packaging and purification

Three shRNA sequences and control siRNA sequence were designed and synthesized by RIBOBIO Co, Ltd (Guangzhou, China). The shRNAs were inserted into a lentiviral vector pSIH1-H1-copGFP. The packaging and purification of lentivirus were executed according to previously reported (38). Briefly, all the plasmids co-transfected into 293T cells and the supernatant was collected and filtered with a 0.45 µm filter 48h after transfection. The transfection efficiency was confirmed using further confirmed by the expression of GFP using fluorescent inverted microscope (Olympus IX83).

### Proliferation and invasion assay

MDA-MB-231 cells, classified into Control shRNA and shRNA groups, were seeded in 96-well plates at a density of 6 × 10³ cells per well and cultured in 100 μL of complete DMEM medium. Cell viability was assessed using the Cell Counting Kit-8 (CCK-8) from Beyotime (Shanghai, China), following the manufacturer’s protocols (39). Additionally, MDA-MB-231 cells were seeded in 24-transwell inserts with 8-µm pores (Coring, NY, USA) for invasion assays. Briefly, the upper surface of the filter was pre-coated with Matrigel (BD Biosciences, USA). Transwell was dried at 37°C for 4 h and placed at room temperature overnight. Cells from different treated groups were added to the upper chamber in 200 µL of serum-free medium, and 500 μL of 10% FBS medium were added to the lower chamber. After 24 h, crystal violet staining was used to visualize the cells that invaded through the filter (40). The images were analyzed using image J software (National Institutes of Health, Bethesda, MD, USA) (41).

### Cytokine array

The culture supernatant from Control shRNA and EPHA2 shRNA groups was collected, and the levels of IL-1β, and IL-18 proteins were quantified using ELISA kits purchased from Shanghai Enzyme-linked Biotechnology (Shanghai, China). Briefly, the prepared samples and standards were added to the wells and incubate at 37°C for 30 minutes. After incubation, the wells were washed to remove unbound substances. Next, the enzyme substrate was added, and incubated again at 37°C for 30 minutes. Then, the stop solution was added, and allowed to incubate for 10 minutes. Finally, the optical density (OD = 450 nm) value was measured after a 15-minute interval, and the results was calculated (42).

### Western blot

Western blot analysis was performed according to our established protocols. The cells samples were harvested and lysed in RIPA lysis solution containing 1% protease inhibitor for 30 min on ice, then centrifuged for 10 min (12,000 rpm, 4°C). Protein concentrations were quantified using BCA method. Then, 20 μg of proteins from each sample were electrophoresed in a 12% SDS-PAGE gel and transferred onto PVDF membranes (Millipore, USA). The membranes were incubated with 5% skim milk for 2 h at 37°C and probed with different primary antibody (Boster Biotechnology, China) overnight at 4°C, followed incubation with secondary antibody (CST, USA) for 2 h at 37°C. After washing the membranes three times with TBST for 5 minutes each time, the protein bands were detected using Tanon Multi imaging system. The protein levels were quantified using ImageJ Software.

### RNA extraction and quantitative real-time PCR experiments

Total RNA was extracted from cells using TRIzol (Invitrogen Technologies, USA). cDNA was synthesized using cDNA Synthesis Kit with genomic DNA removal (Vazyme Biotech Co., Ltd., China) according to the manufacturer’s recommendations. RT-PCR was performed using a SYBR Green mix (Vazyme Biotech Co., Ltd., China) for 40 amplification cycles. The relative expression levels of the target genes in each sample were evaluated using the 2^-△△Ct^ method. β-actin was used as internal reference gene. All qRT-PCR primer sequences used in this study were obtained from PrimerBank. The corresponding PrimerBank IDs for the genes IL18, IL1B, NLRP3, and SLC7A11 are as follows: IL18 (342349317c1), IL1B (27894305c3), NLRP3 (208879435c1), and SLC7A11 (80861465c3).

### Statistical analysis

GraphPad Prism 8 (GraphPad Software, USA) and Origin 2019b software (OriginLab, MA, USA) were used to generate common charts and perform data statistics. Data are presented as mean ± SD. The statistical differences between groups were analyzed using Student’s t test or one-way ANOVA. A *p* value < 0.05 was considered statistically significant.

## Results

### The expression characteristics of EPHA2 in TNBC

**Figure 1:**
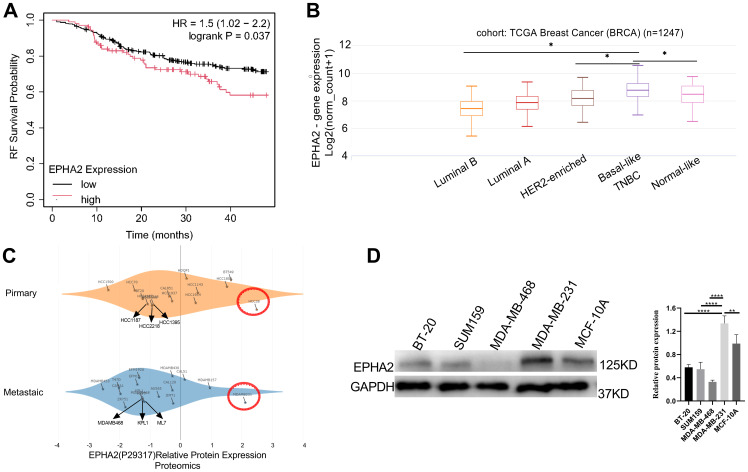
Analysis of EPHA2 expression **(A)** High EPHA2 were associated with shorter relapse-free survival (RFS) in TNBC (Logrank *p* = 0.037) as demonstrated by Kaplan-Meier analysis. **(B)** According to the results of UCSC Xena in different BRCA subtypes, the expression level of EPHA2 was higher in TNBC than that in Luminal A (*p* = 0.034), Luminal B (*p* = 0.019) and normal-like (*p* = 0.031) (Mann–Whitney test). **(C)** Relative protein expression of EPHA2 in different BRCA cell lines derived from metastatic tumors and primary tumors using DepMap portal. **(D)** The confirmation of relative protein expression of EPHA2 in different BRCA cell lines using western blot. *p < 0.05, **p < 0.01, ****p < 0.0001.

Upregulation of ephrin receptor expression enhances various malignant behaviors in cancer cells (43, 44). However, the role of ephrin receptors in cancer development and progression is complex. Our present study focused on the roles of EPHA2 in TNBC. Using Kaplan-Meier plotter, we found that high EPHA2 were associated with shorter relapse-free survival (RFS) in TNBC (**Figure 1A**). Furthermore, we confirmed that EPHA2 expression was higher in Basal like/TNBC, compared to the normal like and other BRCA subtype, according to TCGA Breast Cancer database using UCSC Xena platform (**Figure 1B**). The infiltration of immune cells in the tumor microenvironment may influence the progression and prognosis of various cancers. To investigate this relationship in TNBC, we utilized the Timer 2.0 (TCGA database) to analyze the relationship between EPHA2 expression and immune cell infiltration. Our results showed a positive correlation between EPHA2 expression and infiltration of dendritic cells, and neutrophils (**Supplementary Figures S1A, B**), while negatively correlated with B cells (**Supplementary Figure S1C**). There was no significant correlation between EPHA2 expression and infiltration of CD4+ T cells, and CD8+ T cells, and macrophages (**Supplementary Figures S1D–F**). Additionally, based on DepMap portal, we found that EPHA2 expression in both metastatic and primary tumors was independent of cell line origin. Significantly higher levels were observed in the MDA-MB-231 cell line, which is associated with metastatic TNBC (**Figure 1C**). Western blot analysis further confirmed variations in EPHA2 protein levels among different tumor cell lines (**Figure 1D**), consistent with the bioinformatics analysis shown in **Figure 1C**. Additionally, we compared the differential expression of EPHA2 among various breast cancer cell lines of different subtypes. The results indicated that the TNBC-associated cell line MDA-MB-231 exhibited the highest levels of EPHA2 expression, while lower levels were observed in other breast cancer subtypes (**Supplementary Figure S2**). These findings highlighted EPHA2 is highly expressed in TNBC both *in vivo* and *in vitro*, and is negatively associated with the survival.

### EPHA2 mainly expressed in stromal cells and epithelial cells

**Figure 2:**
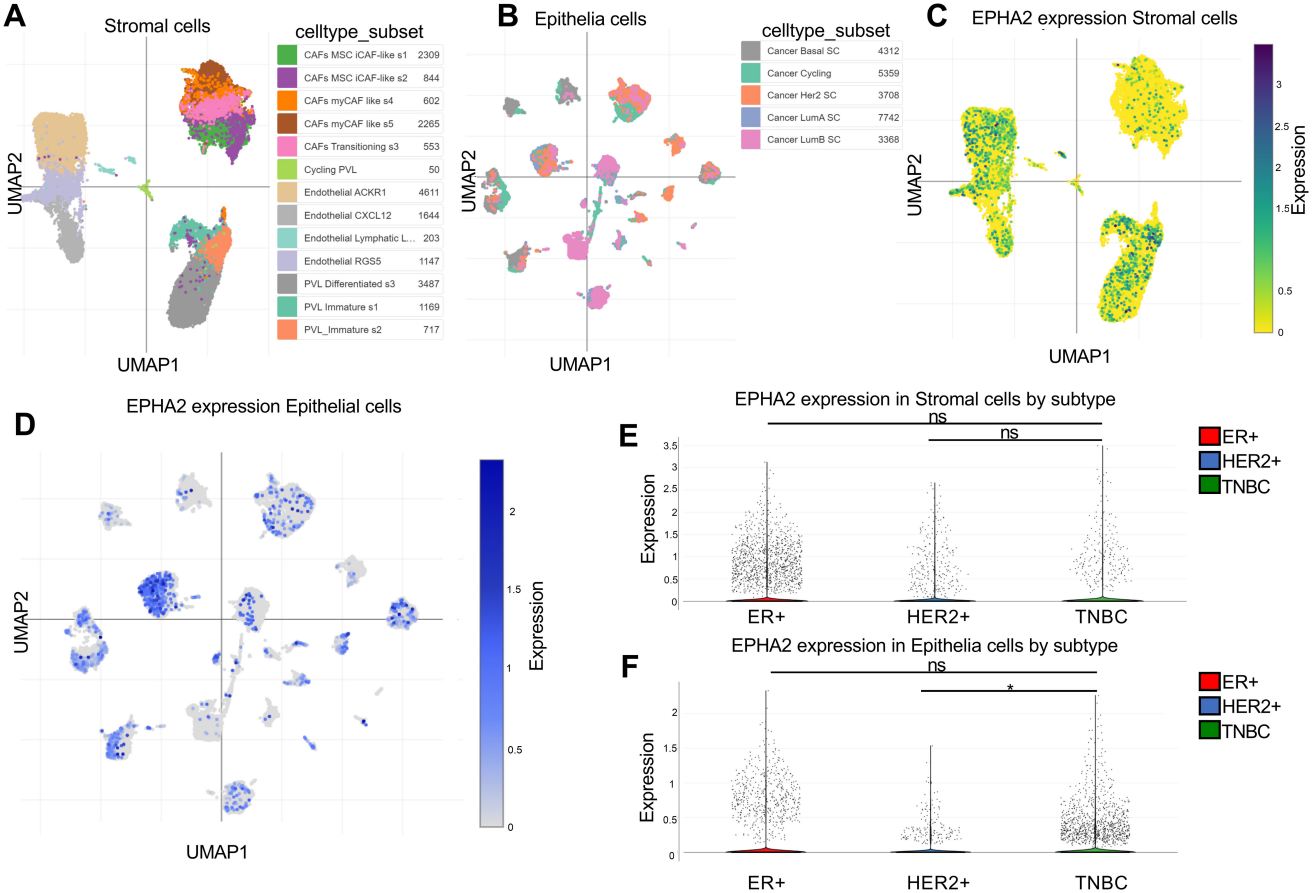
Spatial distribution of cell types expressing EPHA2. **(A)** UMAP plots of stromal cells. **(B)** UMAP plots of epithelial cells. **(C)** EPHA2 expression distribution in stromal cells by subtype and celltype_subset. **(D)** EPHA2 expression distribution in epithelial cells by celltype_subset and subtype. **(E)** EPHA2 expression in different stromal cells. **(F)** EPHA2 expression in different epithelial cells. All these were analyzed using Single Cell Portal (GSE176078). *p < 0.05, ns indicates no significant difference (Mann–Whitney test).

The cellular heterogeneity within the breast cancer tumor microenvironment drives progress in single-cell research and enhances the development of cancer therapeutics. To investigate the expression of EPHA2 at the single-cell level, we analyzed the GSE176078 dataset using the Single Cell Portal. Cells were clustered into distinct types based on specific markers. Our analysis revealed that EPHA2 was predominantly expressed in stromal and epithelial cells, which were further classified into various cell type subsets (**Figures 2A, B**). We assessed the distribution and relative expression of EPHA2 across a diverse array of stromal and epithelial cell type subsets (**Figures 2C, D**). Notably, the number of ER-positive epithelial cells was higher than that of HER2-positive cells. Additionally, the number of TNBC cells in stromal compartments was lower than that in ER-positive cell lines (**Figure 2E**). Conversely, within the tumor microenvironment, the quantity of endothelial cells in TNBC was greater than that in both ER-positive and HER2-positive cells (**Figure 2F**).

These findings suggest that epithelial cells within TNBC tumors play an important role. Further exploration of the functions and roles of EPHA2 in epithelial cell subpopulations will contribute to the advancement of clinical drug development and the formulation of treatment strategies for TNBC.

### EPHA2 knockdown inhibited MDA-MB-231 proliferation and invasion

**Figure 3:**
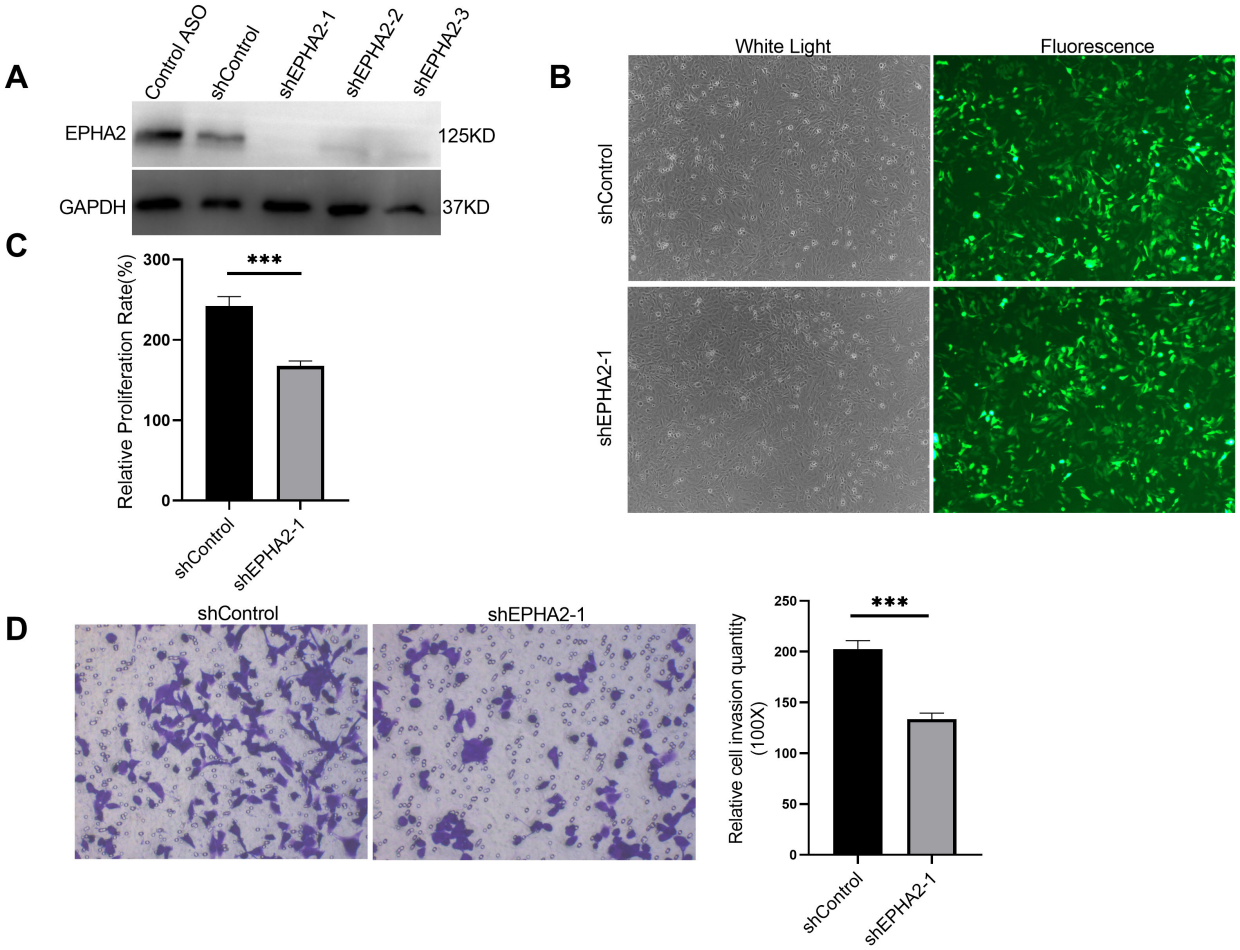
EPHA2 knockdown affected MDA-MB-231 proliferation and invasion. **(A)** The verification of the knockdown efficiency of three EPHA2 shRNAs. **(B)** The transduction efficiency of EPHA2 shRNA was confirmed using Fluorescence microscopy. **(C)** Relative proliferation of MDA-MB-231 cells was decreased in EPHA2 shRNA group, compared to the control shRNA group (*p* = 0.0007, *t*-test). **(D)** Knockdown of EPHA2 inhibited invasion of MDA-MB-231 as assessed by crystal violet staining (*p* = 0.0003, *t*-test). Bars represent the mean ± SD. ***p < 0.001.

To further elucidate the role of EPHA2 in BRCA, we selected TNBC cell line MDA-MB-231, which exhibits a high expression level of EPHA2. We designed three short hairpin RNAs (shRNAs) targeting different sequences of EPHA2 and packaged them into lentiviruses. The efficacy of these shRNAs (shEPHA2-1, shEPHA2–2 and shEPHA2-3) was evaluated using western blot. Results showed that shEPHA2–1 exhibited the highest knockdown efficiency among three shRNAs (**Figure 3A**). Therefore, shEPHA2–1 was used for subsequent investigations. Additionally, both the shControl and shEPHA2–1 exhibited high expression efficiency (**Figure 3B**).

Importantly, we found that the proliferation of MDA-MB-231 cells was significantly inhibited in the shEPHA2–1 group compared to the shControl group (**Figure 3C**), corroborating previously reported findings (4, 45). Furthermore, we assessed the invasion capacity of the cells using a transwell assay, which demonstrated that knockdown of EPHA2 significantly inhibited cell invasion (**Figure 3D**).

### EPHA2 knockdown was associated with pyroptosis in breast cancer

**Figure 4:**
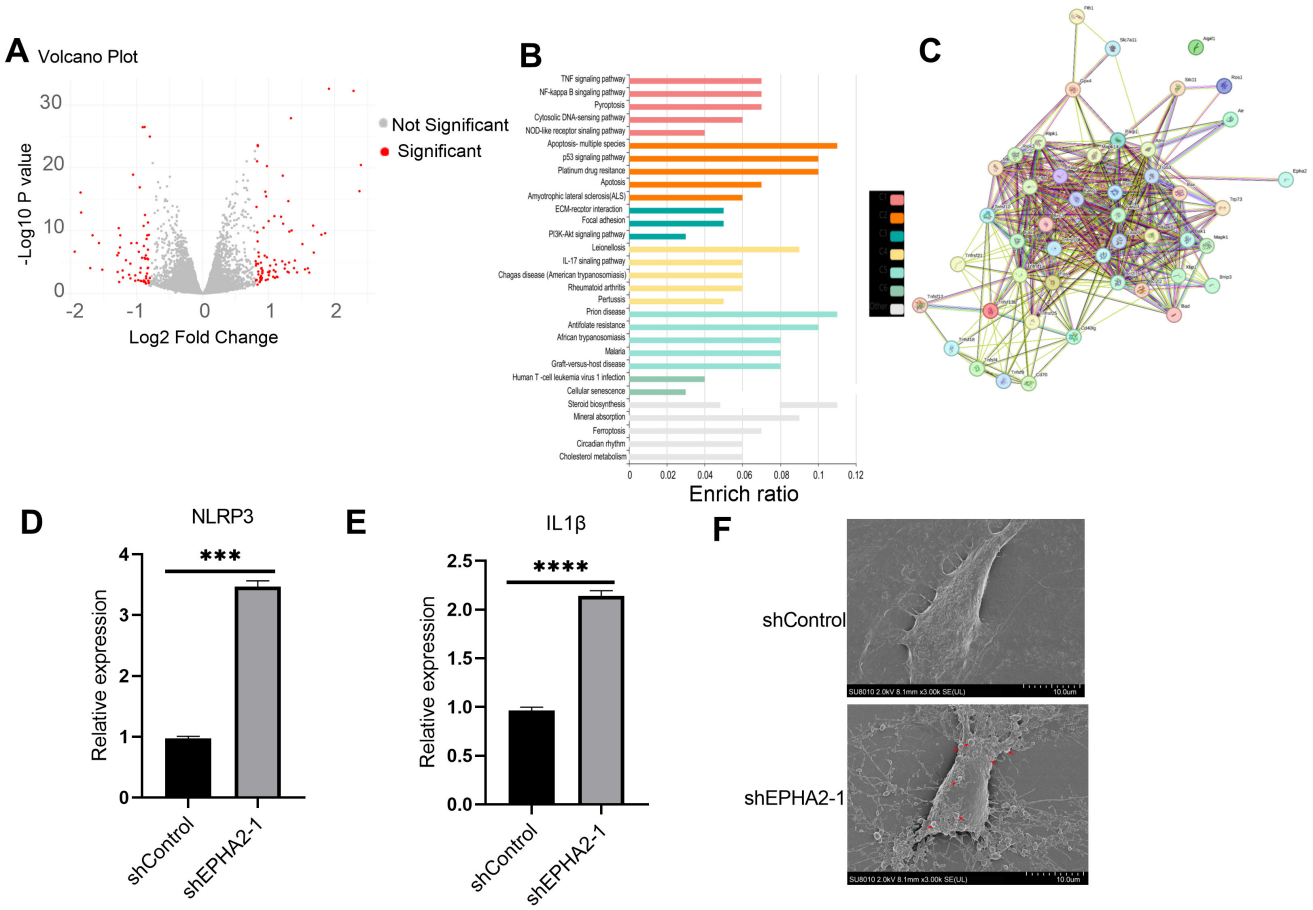
EPHA2 knockdown induced MDA-MB-231 pyroptosis. **(A)** Volcano plot demonstrating an overview of the differential expression of all genes. **(B)** Enrichment ratio and enriched function of these DEGs was analyzed using KOBAS online server. C1-C5 represent different clusters. **(C)** STRING protein-protein interaction network. Proteins are represented as nodes while interactions appear as edges. Relative NLRP3 expression **(D)** and IL1β expression **(E)** in EPHA2 shRNA groups were all lower than in control shRNA groups (*p* = 0.0006 and *p* = 0.0001, respectively, *t*-test). **(F)** The electron microscopy images of control shRNA and EPHA2 shRNA, red arrows indicating the apoptotic bodies for pyroptotic morphology. Bars represent the mean ± SD.

To elucidate the roles of EPHA2 in cancer cell death, we conducted RNA sequencing (RNA-seq) on cells obtained from both the shControl and shEPHA2–1 groups. The resulting volcano plot illustrated the differential gene expression (DGE) between the two groups (**Figure 4A**). Subsequent analysis using the KOBAS online server revealed significant enrichment of the pyroptosis pathway among the differentially expressed genes (**Figure 4B**).

We further examined the interactions between selected pyroptosis-related genes and the DGEs, with the corresponding protein-protein interactions depicted in **Figure 4C**. Specifically, we compared the expression levels of NLRP3 and IL-1β between the control and EPHA2 knockdown groups, and found that EPHA2 knockdown resulted in increased expression of both NLRP3 and IL-1β (**Figures 4D, E**).

Additionally, transmission electron microscopy (TEM) analysis of cell morphology indicated that cells in the EPHA2 knockdown group exhibited typical characteristics of pyroptosis. Inflammatory bodies were also observed in EPHA2 knockdown group (**Figure 4F**).

These findings suggested that EPHA2 played a crucial role in regulating cell death pathways and inflammatory responses in BRCA, highlighting its potential as a key regulator of pyroptosis in this context.

### Analysis of pyroptosis-related proteins and the AKT/PI3K signaling pathway

**Figure 5:**
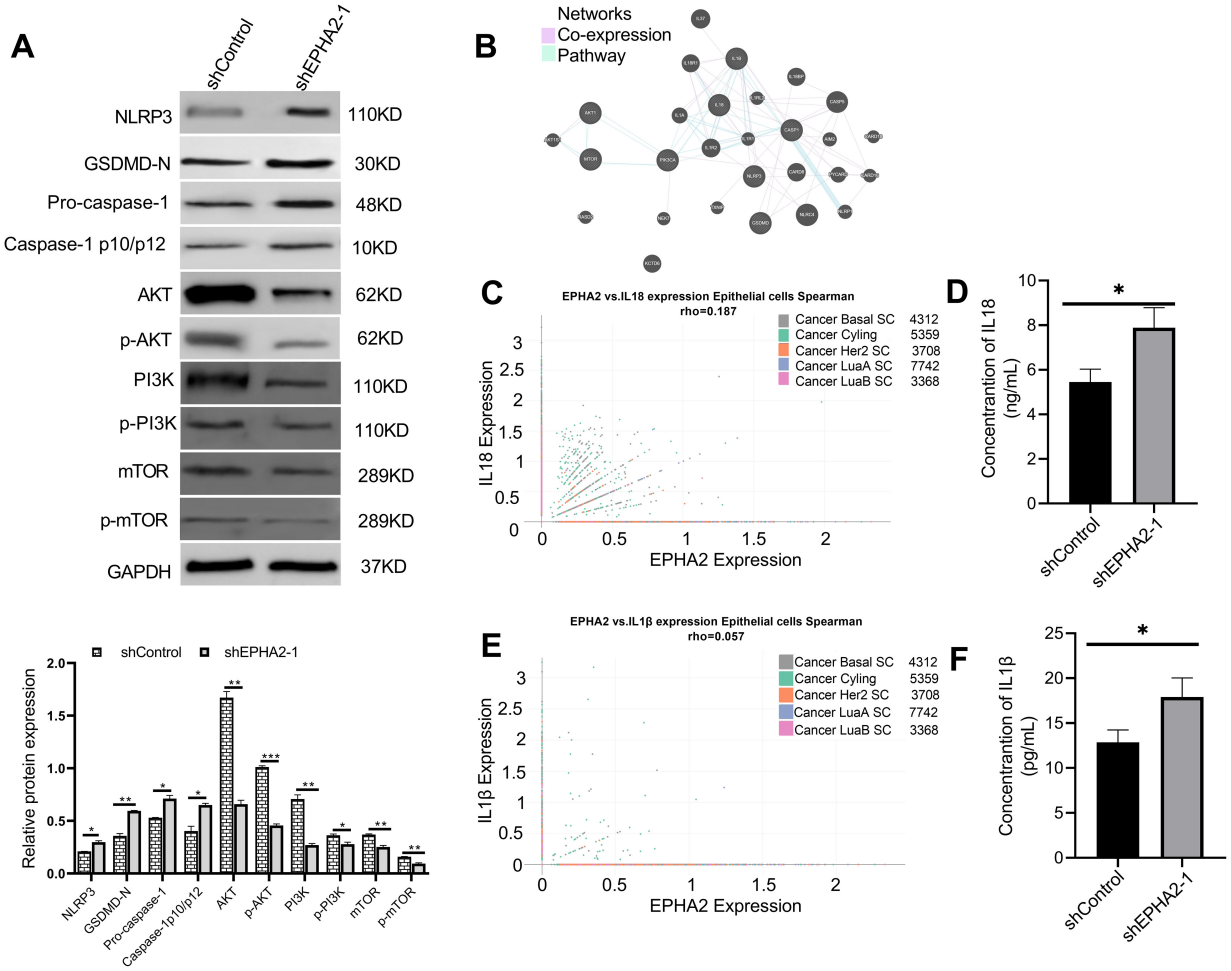
EPHA2 knockdown induced pyroptosis pathway activation and inhibits PI3K/AKT/mTOR signal pathway activation. **(A)** Western blot analysis of NLRP3, GSDMD, caspase-1, AKT, p-AKT, PI3K, p-PI3K, mTOR and p-mTOR expression. *p<0.05, **p < 0.01, ***p < 0.001. Statistical analysis of the two groups were performed using two-tailed Student’s *t* test. **(B)** Analysis of pathways and co-expression data for pyroptosis and genes related to the PI3K/AKT/mTOR signaling pathway using GeneMANIA. **(C)** Correlation of EPHA2 and IL18 expression in epithelial cells by subtype was analyzed based on Single Cell Portal. **(D)** The concentration of IL18 in cell culture supernatant of shEPHA2–1 was lower than in shControl RNA group (*p* =0.0174, *t*-test). **(E)** Correlation of EPHA2 and IL1β expression in epithelial cells by celltype_subset was analyzed based on Single Cell Portal. **(F)** The concentration of IL1β in cell culture supernatant of shEPHA2–1 was lower than in shControl RNA group (*p* =0.0259, *t*-test). Bars represent the mean ± SD.

Pyroptosis-related proteins have been identified and evaluated in various studies (46). Notably, genes within the AKT/PI3K signaling pathway were found to be enriched (**Figure 4B**). To elucidate the relationship between pyroptosis and the AKT/PI3K signaling pathway, we analyzed proteins associated with pyroptosis as well as the phosphorylation of AKT, PI3K, and mTOR using western blot. The results indicated an upregulation of NLRP3, along with the cleavage of GSDMD and pro-caspase-1, confirming the identification of GSDMD and pro-caspase-1 (**Figure 5A**). Additionally, we observed decreased levels of phosphorylated AKT (p-AKT), PI3K (p-PI3K), and mTOR (p-mTOR) (**Figure 5A**) in shEPHA2–1 group, compare to the control group.

We employed GeneMANIA to construct interaction networks, selecting two parameters: co-expression and pathway (**Figure 5B**) (47). Based on the above results, we analyzed the correlation between EPHA2 expression and the expression of IL-1β and IL-18 in epithelial cells using Single Cell Portal platform. The correlation analysis revealed that the mRNA expression levels of EPHA2, IL-1β, and IL-18 were not statistically significant, as the Spearman correlation coefficient was less than 0.3 (**Figures 5C, E**).

Furthermore, we collected culture supernatants from both control shRNA and EPHA2 shRNA groups and measured the concentrations of IL-18 and IL-1β using the ELISA method. We found that the concentrations of IL-18 and IL-1β were significantly higher in the EPHA2 knockdown group compared to that in the control group (**Figures 5D, F**). Additionally, we measured the release of lactate dehydrogenase (LDH), which is frequently used as an indicator of pyroptotic cell cytotoxicity. Our results showed that LDH release significantly increased in shEPHA2–1 group than that in the control shRNA group (**Supplementary Figure S3**)

These data indicated that the knockdown of EPHA2 induced pyroptosis in MDA-MB-231 cell line, while inhibited the AKT/PI3K signaling pathway.

To further confirm that EPHA2 affects pyroptosis through the PI3K/AKT/mTOR pathway, we used an agonist of the PI3K/AKT signaling pathway, recilisib. The results indicated a significant decrease in pyroptosis-associated proteins in the PI3K/AKT agonist group (shEPHA2 + agonist), compared to the EPHA2 knockdown group (**Supplementary Figure S4**). The finding further confirms that EPHA2 regulates pyroptosis through the AKT/PI3K signaling pathway in TNBC.

### Association among EPHA2, pyroptosis, AKT/PI3K signaling pathway and ferroptosis

**Figure 6:**
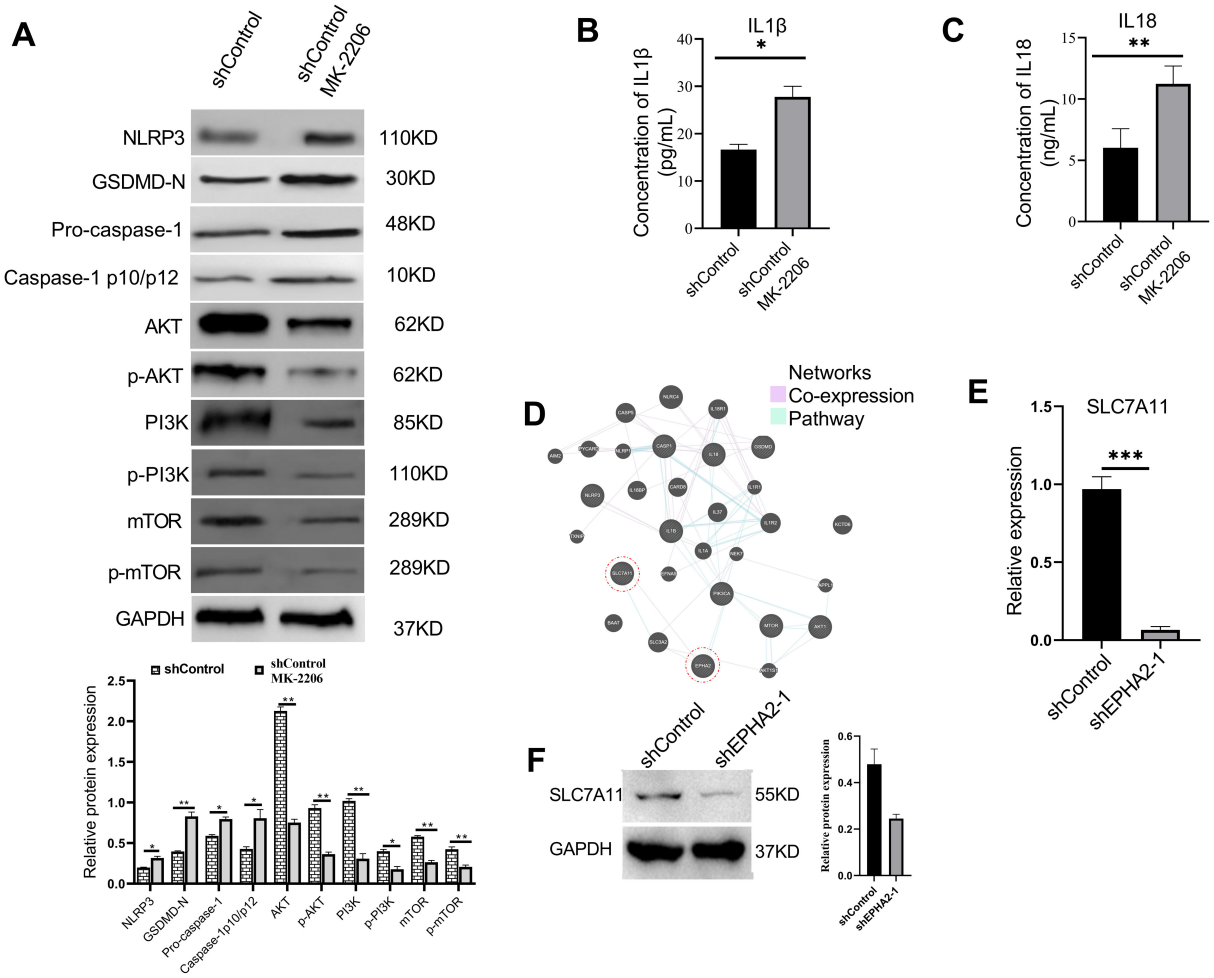
AKT inhibition induced MDA-MB-231 pyroptosis and decrease SLC7A11 expression. **(A)** Western blot analysis of NLRP3, GSDMD, caspase-1, AKT, p-AKT, PI3K, p-PI3K, mTOR and p-mTOR expression in MDA-MB-231 cells treated with AKT inhibitor or control buffer. *p<0.05, **p < 0.01. Statistical analysis of the two groups were performed using two-tailed Student’s *t* test. The concentration of IL1β **(B)** and IL18 **(C)** in cell culture supernatant were all significantly elevated in the MK-2206-treated group, compared to the control group (*p* = 0.0134 and *p* = 0.0015, respectively, *t*-test). **(D)** Analysis of pathways and co-expression data of SLC7A11, EHPA2, pyroptosis related genes and AKT/PI3K/mTOR related genes. Relative mRNA expression levels (*p* = 0.0002, *t*-test) **(E)** and protein expression levels **(F)** of ferroptosis-associated gene SLC7A11 were all decreased in shEPHA2–1 group. Bars represent the mean ± SD.

Knockdown of EPHA2 has been observed to trigger pyroptosis, while concurrently inhibited the AKT/PI3K signaling pathway. To investigate the roles of EPHA2 in modulating pyroptosis in MDA-MB-231 cells via the AKT/PI3K pathway, we employed MK-2206, an AKT inhibitor. Our results demonstrated a significant increase in the expression levels of NLRP3, GSDMD, and pro-caspase-1, alongside a marked inhibition of the AKT/PI3K signaling pathway in MK-2206 group, compared to control group (**Figure 6A**). Additionally, we measured the release of lactate dehydrogenase (LDH), which is frequently used as an indicator of pyroptotic cell cytotoxicity. The results demonstrated that the AKT inhibitor significantly increased LDH release compared to the control shRNA group (**Supplementary Figure S5**). These findings indicate that the AKT inhibitor exhibits a similar trend in pyroptosis as observed with EPHA2 knockdown.

Furthermore, compared to the control group, the concentrations of pro-inflammatory cytokines IL-1β and IL-18 were significantly elevated in the MK-2206-treated group (**Figures 6B, C**). Analysis of our transcriptional data, combined with the previously reported GEO dataset (GSE141880), revealed significant alterations in the expression of over 40 members of the SLC family. We subsequently constructed a network that interlinks SLC7A11, EPHA2, pyroptosis-related genes, and genes associated with the AKT/PI3K signaling pathway (**Figure 6D**). Furthermore, we found that knockdown of EPHA2 led to a decreased expression of SLC7A11 both in mRNA (**Figure 6E**) and protein levels (**Figure 6F**), a phenomenon also observed following AKT inhibition.

## Discussion

In this study, we investigated the expression and functional implications of EPHA2 in TNBC based on bioinformatics, transcriptome and western blot. Our findings confirmed previous studies that have highlighted the complex role of ephrin receptors in promoting malignant behaviors, including proliferation and invasion, yet underscore the variability in their expression profiles (4, 5). Furthermore, our results suggested elevated EPHA2 was associated with shorter survival.

Reanalysis of existing single-cell datasets, we observed that the predominant expression of EPHA2 in stromal and epithelial cells corroborates previous findings regarding its role in the tumor microenvironment (35, 36). Notably, our identification of EPHA2 knockdown leading to increased pyroptosis aligns with studies suggesting that ephrin signaling can impact cell death pathways. However, our research extends this understanding by explicitly linking EPHA2 to the AKT/PI3K signaling pathway and demonstrating that its inhibition can enhance pyroptotic responses in breast cancer cells, a relationship that has not been thoroughly explored in prior research.

Recently, there has been significant clinical interest in combining targeted therapies with immune checkpoint inhibitors (ICI) to manipulate the immune setpoint (48). Increasing evidence suggests that Eph/ephrin signaling in various cancer plays an important role in promoting immunosuppression within the tumor microenvironment (TME). Targeting the Eph receptor signaling in conjunction with immune checkpoint inhibitors has been proposed as a promising approach in cancer immunotherapy research (49). Additionally, it has been reported that PD-L1 inhibitors, in combination with chemotherapy or radiotherapy, can eradicate cancer cells by inducing pyroptosis (50). Our findings indicated that EPHA2 was involved TNBC progression by regulating pyroptosis. These results indicate that EPHA2 cooperates with ICI to enhance tumor immunogenicity via pyroptosis, which may be a potential therapeutic strategy for TNBC.

EPHA2 emerges as a critical regulator of tumor cell death, with roles that are context-dependent and tumor-specific. Apoptosis is a programmed cell death pathway critical for maintaining tissue homeostasis and suppressing tumor growth. Han et al. demonstrated that exosomal EPHA2 from highly metastatic breast cancer cells promotes angiogenesis and suppresses apoptosis by activating the Ephrin A1-EPHA2 forward signaling and the AMPK pathway (51). Mubthasima et al. showed that EPHA2 contributes to mitochondrial dynamics, autophagy, and mitophagy in cervical cancer, enhancing sensitivity to cisplatin by modulating inflammatory programmed cell death pathways (52, 53). This study presents several innovative aspects, particularly the elucidation of the interplay between EPHA2, SLC7A11, and pyroptosis within the context of the AKT/PI3K pathway. The USP4/CARM1 axis was shown to increase SLC7A11 expression, promoting malignant transformation and resistance to ferroptosis (54). Cao et al. identified the PRMT1/SLC7A11 axis as a critical pathway for inhibiting ferroptosis in colorectal cancer, providing a targetable vulnerability (55). Pyroptosis is often triggered by ROS, which EPHA2 indirectly influences. Both EPHA2 knockdown and AKT inhibition resulted in decreased SLC7A11 expression at mRNA and protein levels. This indicates a regulatory network in which SLC7A11 acts as a downstream target of the AKT/PI3K pathway, contributing to the suppression of ferroptosis. However, a limitation of this study is its reliance on a single TNBC cell line, which may not fully capture the heterogeneity observed in clinical settings. Future research should expand to include a broader range of cell lines and *in vivo* models to validate these findings. Additionally, exploring the mechanistic pathways through which SLC7A11 mediates these effects could provide deeper insights into its role in cancer biology.

The implications of our findings are significant for future research and clinical practice. By elucidating the role of EPHA2 and its interaction with key signaling pathways, this study paves the way for potential therapeutic strategies targeting these mechanisms in breast cancer. Understanding how SLC7A11 regulates pyroptosis could inform new approaches to enhance treatment efficacy, particularly in aggressive subtypes such as TNBC.

Overall, our findings illuminated the dual roles of EPHA2 in TNBC, influencing both tumor progression and cell death pathways. Mechanistically, EPHA2 regulates TNBC progression by suppressing pyroptosis via the AKT/PI3K/mTOR pathway (**Supplementary Figure S6**). Ultimately, our research contributes to a more nuanced understanding of breast cancer biology, with the potential to guide future therapeutic developments and improve patient outcomes.

## Data Availability

The sequencing data have been deposited in the Sequence Read Archive (SRA), under the asscession number PRJNA1207249 (https://www.ncbi.nlm.nih.gov/bioproject/PRJNA1207249).

## Supplementary material

Supplementary Figure 1 The relationships between members of EPHA2 and immune cell infiltration in TNBC. The effects of EPHA2 on the infiltration of dendritic cells **(A)**, neutrophils **(B)**, B cells **(C)**, CD4+ T cells **(D)**, CD8+ T cells **(E)** and macrophage **(F)** were analyzed using TIMER 2.0.

Supplementary Figure 2 The analysis of relative protein levels of EPHA2 among various breast cancer cell lines of different subtypes using western blot.

Supplementary Figure 3 LDH activity was increased in shEPHA2–1 group, compared to the shControl group. **** *p*<0.0001, *t*-test. Bars represent the mean ± SD.

Supplementary Figure 4 AKT agonist reversed the blocking of pyroptosis induced by EPHA2 knockdown in MDA-MB-231. *** p*<0.01, *t*-test. Bars represent the mean ± SD.

Supplementary Figure 5 LDH activity in shControl and shControl+MK-2206 groups. **** *p*<0.0001, *t*-test. Bars represent the mean ± SD.

Supplementary Figure 6 Schematic diagram illustrating the potential molecular mechanisms through which EPHA2 was involved in TNBC progression by regulating pyroptosis via the AKT/PI3K/mTOR pathway.
