# Supplementary figures and images for: EPHA2 promotes triple-negative breast cancer progression by suppressing pyroptosis via the AKT/PI3K/mTOR pathway
Source: Front Oncol. 2025 Aug 22;15:1620122. doi: 10.3389/fonc.2025.1620122 (PMC12411449; doi:10.3389/fonc.2025.1620122)

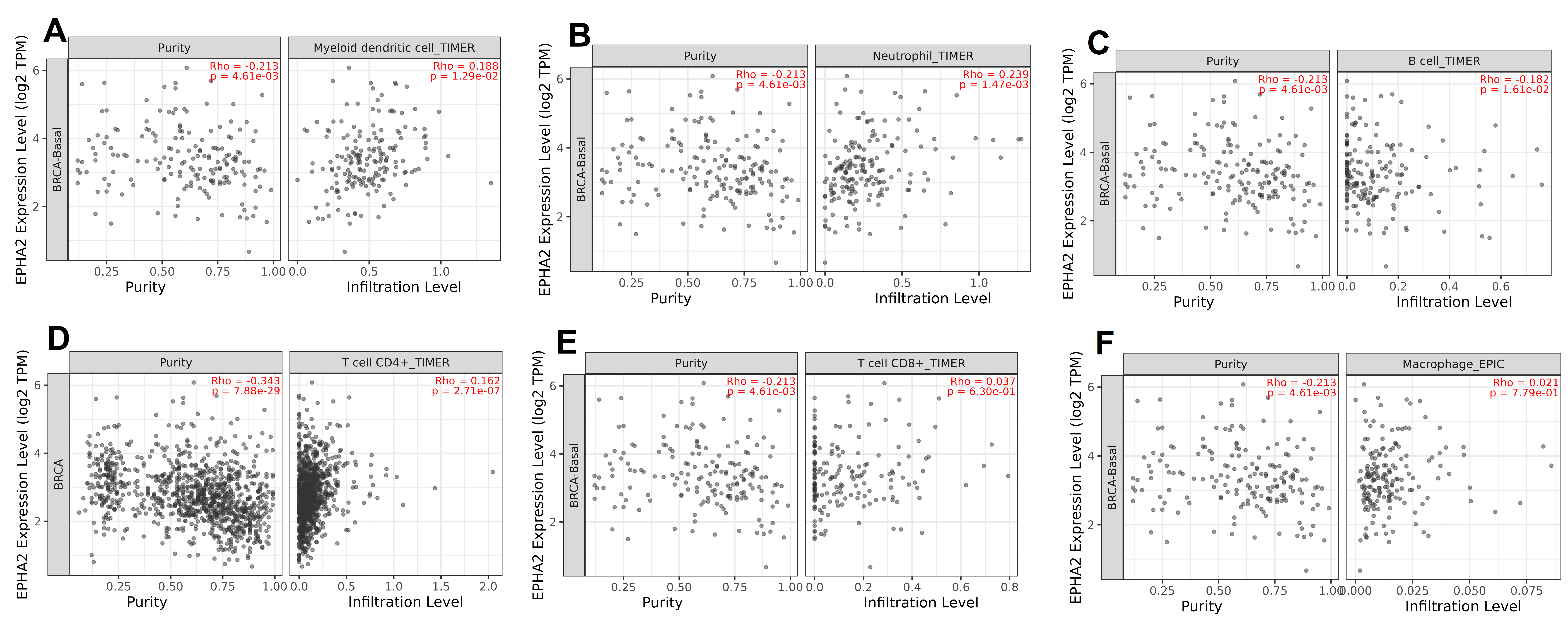

Supplement: Supplementary Figure 1 — The relationships between members of EPHA2 and immune cell infiltration in TNBC. The effects of EPHA2 on the infiltration of dendritic cells (A), neutrophils (B), B cells (C), CD4+ T cells (D), CD8+ T cells (E) and macrophage (F) were analyzed using TIMER 2.0. [file Image1.tif]

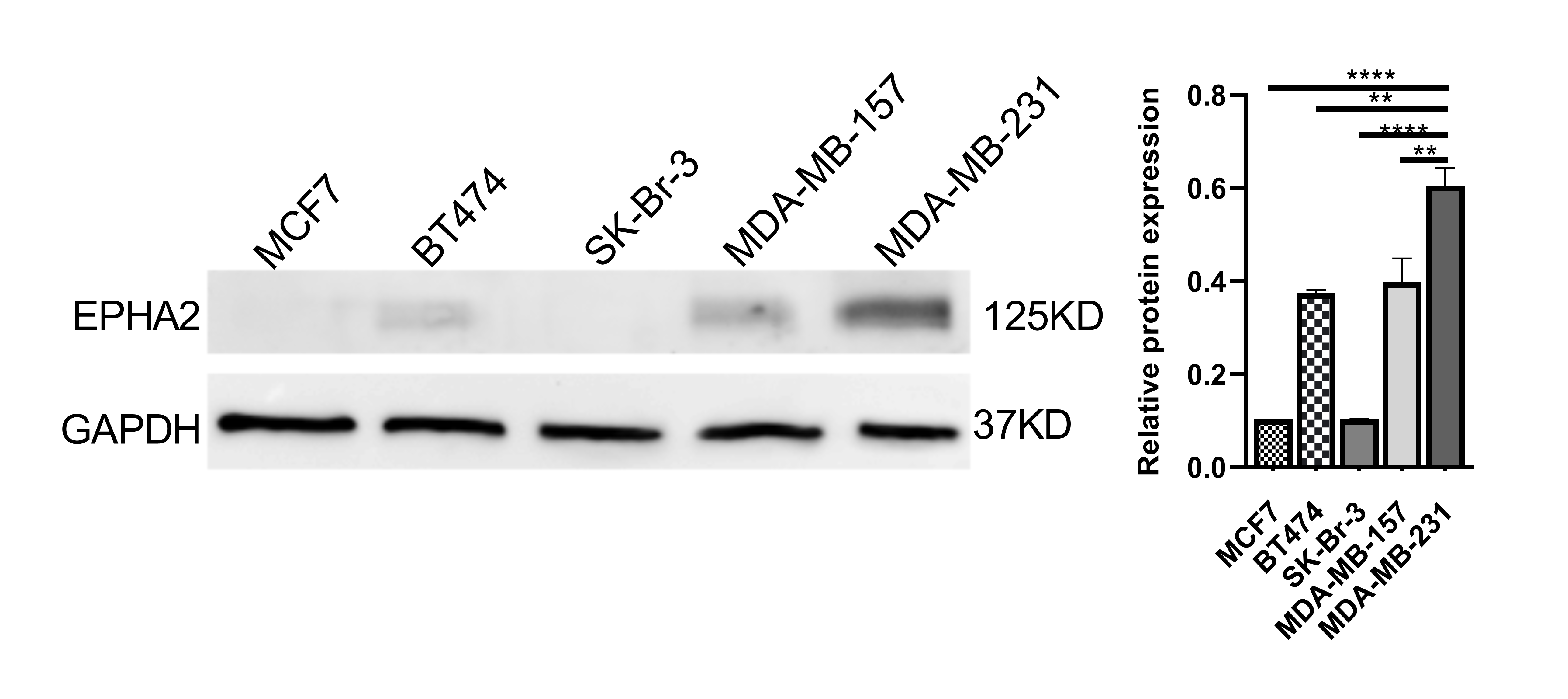

Supplement: Supplementary Figure 2 — The analysis of relative protein levels of EPHA2 among various breast cancer cell lines of different subtypes using western blot. [file Image2.tif]

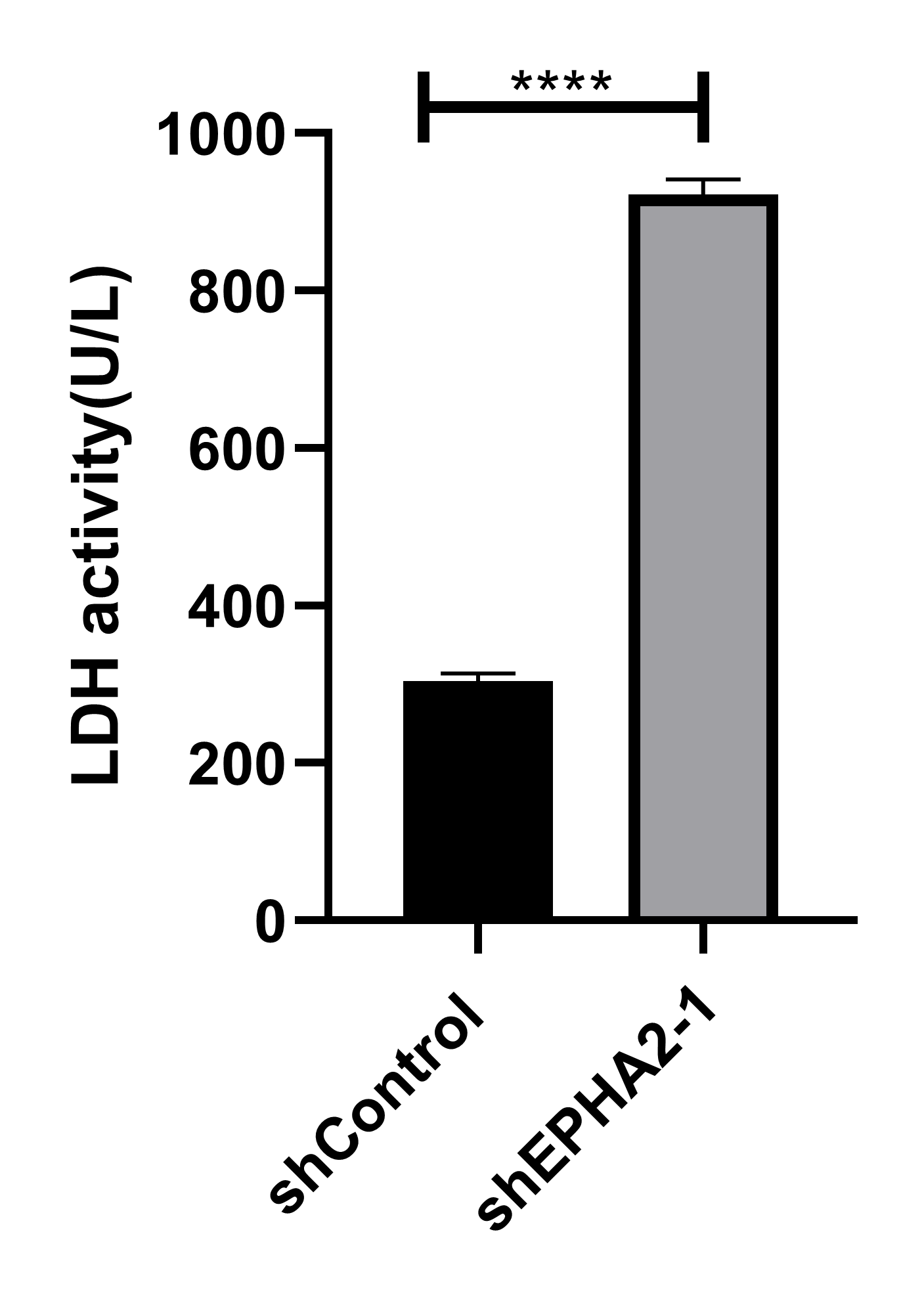

Supplement: Supplementary Figure 3 — LDH activity was increased in shEPHA2–1 group, compared to the shControl group. **** p<0.0001, t-test. Bars represent the mean ± SD. [file Image3.tif]

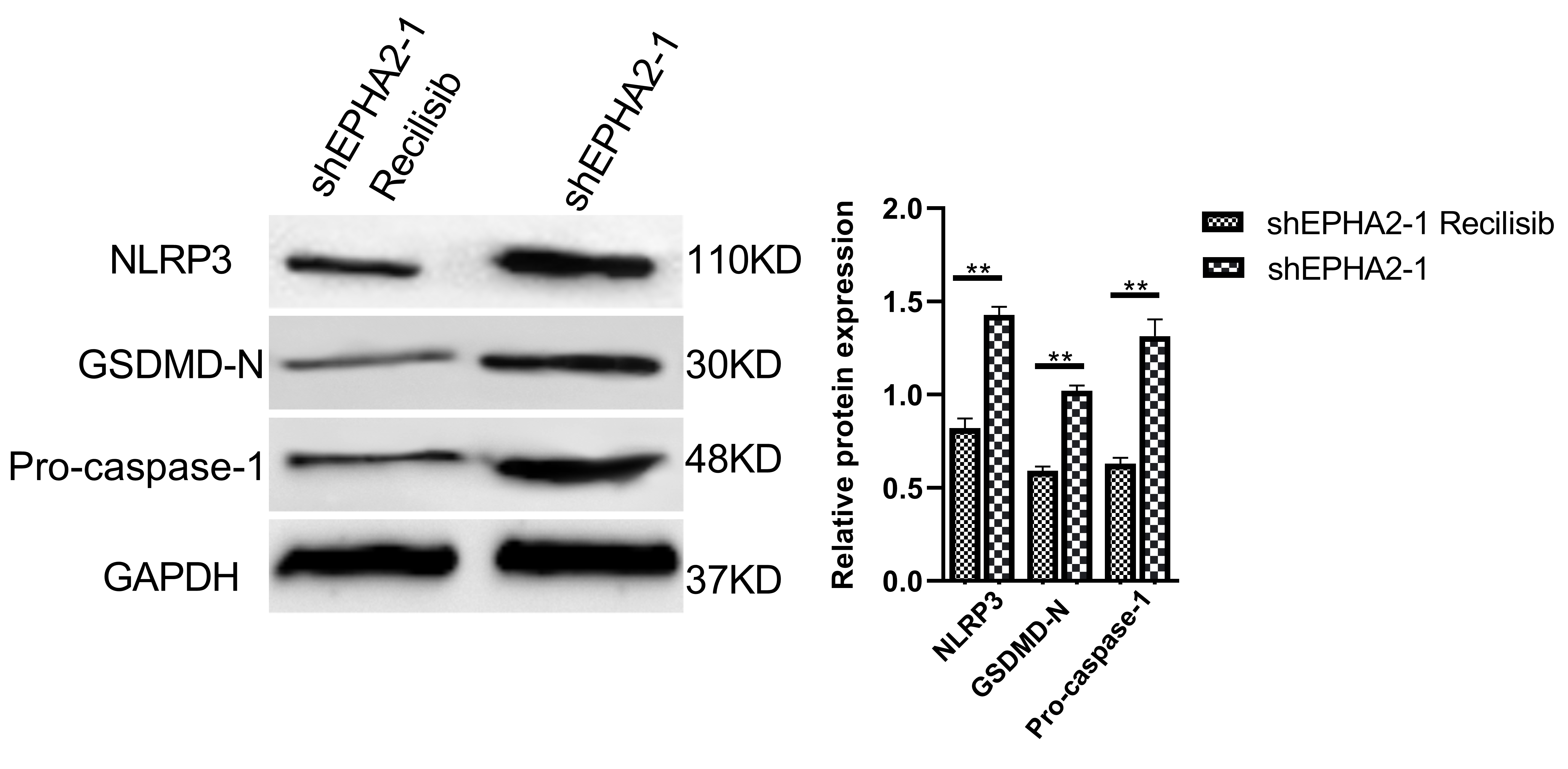

Supplement: Supplementary Figure 4 — AKT agonist reversed the blocking of pyroptosis induced by EPHA2 knockdown in MDA-MB-231. ** p<0.01, t-test. Bars represent the mean ± SD. [file Image4.tif]

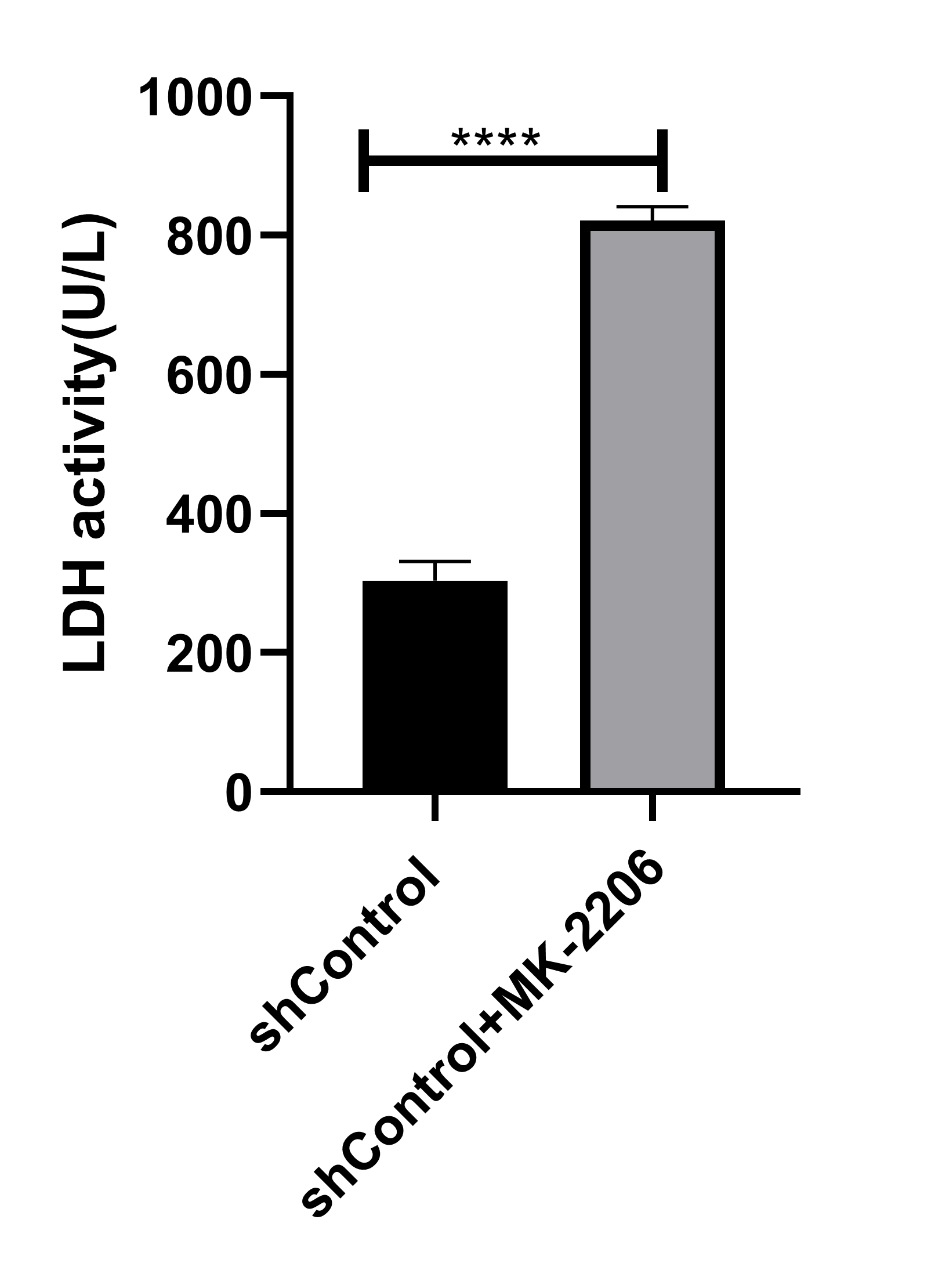

Supplement: Supplementary Figure 5 — LDH activity in shControl and shControl+MK-2206 groups. **** p<0.0001, t-test. Bars represent the mean ± SD. [file Image5.tif]

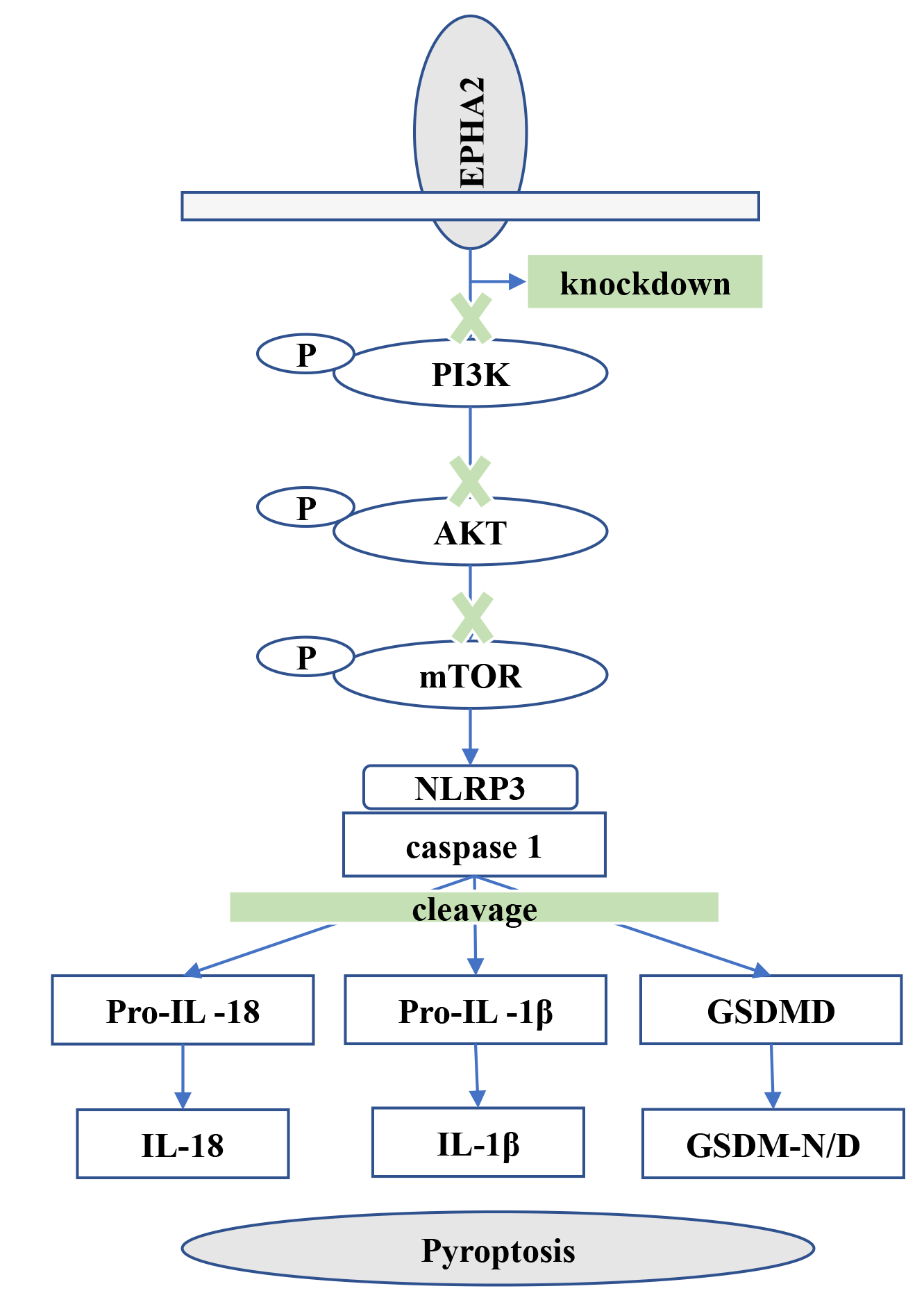

Supplement: Supplementary Figure 6 — Schematic diagram illustrating the potential molecular mechanisms through which EPHA2 was involved in TNBC progression by regulating pyroptosis via the AKT/PI3K/mTOR pathway. [file Image6.tif]
